# Harnessing community-based one health interventions implementation beyond Mpox outbreak management in Africa: insights and benefits

**DOI:** 10.1186/s40249-025-01348-y

**Published:** 2025-09-08

**Authors:** Ernest Tambo, Brice G. Djopmo, Joelle N. Djamfa, Leonel D. Z. Temomo, Odile Djouka, Florence Akiiki Bitalabebo, Jules C. Assob

**Affiliations:** 1 Institute for Global Health Equity, University of Global Health Equity, Butaro, Rwanda; 2 Africa Disease Intelligence, Preparedness and Response, Yaounde, Cameroon; 3 Faculty of Medicine and Pharmaceutical Sciences, University of Douala, Douala, Cameroon; 4 Faculte de Medicine, Universite Des Montagnes, Banekane, Cameroon; 5 The Godley-StGoar Department of Community Health, University of Global Health Equity, Butaro, Rwanda; 6 Department of Public Health, University of Buea, Buea, Cameroon

**Keywords:** Mpox, Outbreak, Public health emergency, Community, Resilience, Recovery, Response, Health security, Africa

## Abstract

**Background:**

Little is documented on key community-based One Health (OH) approach implementation, pro-activeness and effectiveness of interactions and strategies against Mpox outbreak public health emergency in international concern (PHEIC) in various African countries in order to stamp out the persisting Mpox outbreak threat and burden. Prioritizing critical community-based interventions and lessons learned from previous COVID-19, Mpox, Ebola, COVID-19, Rift Valley Fever and Marburg virus outbreaks revealed critical shortcomings in funding, surveillance, and community engagement that plague public health initiatives across the continent. The article provides critical insights and benefits of community-based One Health approaches implementation against Mpox outbreak management in Africa.

**Main body:**

Our findings provides a comprehensive community and primary healthcare systems strategies essential to foster community engagement and resilience, while addressing the social determinants of health. Investing in targeted, effective and contextual community-based OH strategies implementation shows to improve immediate vulnerable communities integrated (human,animal and environment) preparedness and response and building sustainable resilience strategies against Mpox and future emergencies threats. The importance of global and regional multi-sectorial collaboration, solidarity and coordination cannot be over-emphasized, to mobilize resource, sharing knowledge and successes in enhancing local OH anticipatory and ownership programs capabilities for equitably shared benefits. Timely strengthening community empowerment and national health systems last miles, WASH and vaccination activities are essential to control, contain and sustainable recovery from the ongoing Mpox outbreak and future crises. Tackling survivors and at high risk affected populations stigma, fear and misinformation surrounding Mpox those hinder effective health communication and disease management, highlighting the need for culturally sensitive educational and empowerment strategies. A comprehensive assessment community-based one-health approaches implementation was performed to understand and prioritize key data-driven community-based OH strategies in infectious disease outbreaks beyond. Leveraging on outbreak valuable lessons learned and emerging technologies benefits in addressing the health social determinants, optimizing Mpox PHEIC implemented programs capabilities efforts, building communities resilience and sustainable solutions, and prioritizing strategies against outbreaks/pandemics threats and burden.

**Conclusions:**

Catalyzing evidence-based community-based OH governance, leadership and domestic financing commitment serve as a critical engine connecting all stakeholders in prioritizing and optimizing unprecedented outbreaks threats preparedness and response initiatives implementation and upholding global health security returns.

**Graphical Abstract:**

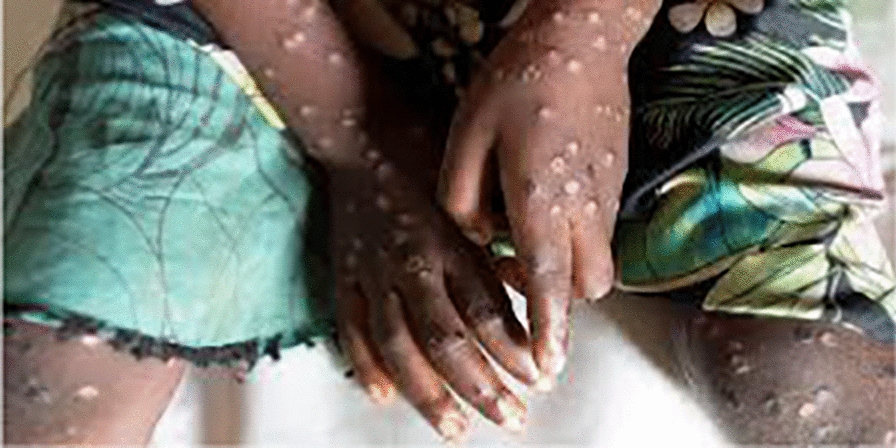

## Background

Mpox outbreak in Africa was declared as a Public Health Emergency of International Concern (PHEIC) by Africa Centre for Disease Control and World Health Organisation (WHO) on August, 2024 calls for an urgent need to assess monkeypox (Mpox) virus geo-diversity and complexity from regional to global health security threat and burden [1]. Initially endemic to Central and West Africa, Mpox resurgence and rising number of cases and deaths reported outside its traditional hotspots has attracted global attention since early 2022, Mpox spread across multiple continents, affecting thousands of individuals [2, 3]. Mpox was declared as a PHEIC in 2022, and was lifted in 2023 as reported cases declined by the WHO [1]. A total 103,048 laboratory-confirmed cases reported as of August 2024, highlighting the urgent need for enhanced public health surveillance and intervention strategies [4]. Admist, 19 WHO-member states across Africa have reported Mpox cases including The Democratic Republic of the Congo, Burundi, Cote d’Ivoire, South Africa, Rwanda, Cameroon, Nigeria and the Central African Republic accounting for most laboratory confirmed cases and reported deaths [1, 2].

It is documented that knowledge gaps in Mpox clade severity and reservoirs, coupled with insufficient financial commitment, have hampered building Mpox preparedness and resilience, while weak Mpox diagnostic and surveillance infrastructures and lack of safety vaccination coverage have delayed timely responses to current outbreaks. In recent years, Mpox has transitioned from a relatively obscure zoonotic infection to a critical global public health concern [1, 2].

Mpox is historically viewed as a rare infection; however, it has been viewed as epidemiological similarities with smallpox and its potential to cause outbreaks in human populations [4, 5]. While the case fatality rate of Mpox is generally lower than that of smallpox eradicated in the 1980 s, WHO estimates suggest increased sub clade severity and case fatality rates (CFR) among vulnerable groups such as sex workers and men that have sex with men(msm), children, aged people, and immuno-deficient individuals [6]. The ongoing subtype clade IIb Mpox outbreak has caused over 100,000 laboratory confirmed cases in 122 countries including 115 countries where Mpox was not previously reported. Both clade I and II Mpox was only reported nationwide in Cameroon and reported travel associated clade I cases in United Kingdom, Germany, Sweden, Thailand, and India [4, 6].

The resurgence of Mpox is influenced by overlapping factors, such as global travel, urbanization, changes in human-animal contact, and environmental shifts exacerbated by climate change and conflict effect, poor understanding of herb immunity and varied pathophysiology manifestations [2]. These transmission dynamics underscore the importance of understanding Mpox not only as a public health challenge, but also as a complex interplay of animal, environmental and ecological to socioeconomic factors [5]. The clinical presentation of Mpox is variable based on subclade I and II, and can mimic that of other infectious diseases, thus complicating diagnosis and treatment.

Interestingly, recent data have established that the manifestations of the illness may differ significantly depending on factors such as age, sex, and immunological status. It highlights the need for clinicians to recognize the range of possible symptoms and remain alert for atypical clinical presentations [6, 7]. Furthermore, social stigma and discrimination around Mpox infection have had negative implications for disclosure, healthcare seeking behaviour, and community engagement and resilience building among affected populations in Africa and globally [1, 8]. Hence, efforts should be devoted to mitigate misconceptions and misinformation about vaccine and vaccination acceptance, barriers to uptake and encourage vulnerable population trust and confidence to vaccination benefits.

Little is documented on key community-based One Health approach implementation in building and scaling up effective and sustained pro-activeness and participatory emergency response strategies against Mpox outbreak PHEIC in various African countries. This approach has shown to be crucial in building integrated and coordinated efforts to quell the persisting Mpox threat and burden amongst vulnerable communities in Africa and globally [3, 5, 7, 9].

The short communication highlights actionable insights and challenges of community-based One Health Mpox outbreak interventions implementation challenges to decision-makers, implementers, in empowering and engaging vulnerable communities. Also, strengthening local and national health systems through integrated, effective and timely One Health Community of Practice, and resilience approaches to accelerate post-Mpox PHEIC outbreak recovery programs to quell the ongoing local, national and continental Mpox outbreak in most affected countries.

## MPXV outbreak in Africa and worldwide

Our findings reported a total of 106,310 Mpox virus laboratory confirmed cases and 234 deaths, whereas in Africa recorded a total of 45,652 confirmed cases and 1492 deaths thus a case fatality rate (CFR) of 3.3% between January 2022 and August 2024 [1]. Mpox outbreak in Africa represents a significant challenge that underscores the complexities of public health, and socioeconomic challenges. The resurgence of Mpox in 2024 highlights the knowledge gaps and weak community and primary healthcare infrastructure compounded by underfunding and inadequate local and regional emergency preparedness and response interventions to unprecedented public emergencies crises [1, 2]. The epidemiological landscapes of African nations have evolved with urban migration, conflict-linked displacement and population increases intensifying the interactions between humans and wildlife, thereby enhancing the potential for zoonotic spillovers [2, 3]. This aqueduct of socioeconomic instability, exacerbated by climate change, has further compounded the vulnerability of communities, particularly in densely populated urban centers where healthcare resources are already stretched thin [4]. As of mid-2024, the number of confirmed cases has illuminated disparities not only in epidemiological data, but also in the capabilities of health systems to respond effectively. For instance, Democratic Republic of Congo and Burundi, Nigeria and South Africa have continued to report substantial numbers of infections, revealing systemic issues related to healthcare access, disease transmission and dynamic, weak community literacy and culture to public intervention trust and adherence, stigma and complexity of Mpox virus clade severity and weak accurate of epidemiological data related to resource and timely interventions implementation challenges (Table 1).

**Table 1:** Total number of cumulative laboratory-confirmed Mpox cases and deaths reported by WHO Region, from 1 January 2022 to 30 September 2024

WHO Region (most affected countries)	Total confirmed cases	Total deaths among confirmed cases	New cases reported in July, 2024	New cases reported in August 2024	New cases reported in Sept. 2024	Fatality rate (per/1000)
Africa (Democratic Republic of Congo, Burundi, Rwanda, Cote d,Ivoire, South Africa Guinea, Central Africa Republic, Nigeria, Cameroon)	64 879	35	175	447	207	8.27
United States of America	27, 965	141	100	151	255	2.24
European Union	7,662	10	567	1, 016	1 298	0.36
Western Pacific	3,979	10	81	163	274	2.86
Southeast Asia (Australia)	956	11	11	15	16	11.89
Eastern Mediterranean	869	1	0	9	2	1.05
Total	106, 310	208	934	1,801	2, 082	

Source: WHO, September 2024. *WHO* World Health Organization

## Integrating community-based one health approach in health systems against zoonotic outbreak in Africa

One Health community-based approaches require engaging and empowering local vulnerable communities in identifying, co-participating and co-implementing integrated and tailored human-animal-environment surveillance and detection, zoonotic outbreak prevention and control measures and interventions, addressing antimicrobial resistance and managing other health issues. Strengthening OH transnational and cross-sectorial partnership and collaborative efforts has emerged and requires building integrated OH surveillance and monitoring systems for early risk detection, timely risk communication and mitigation strategies prior to, during and after zoonotic outbreaks. A evidence community-based OH approach coordinates efforts across human-animal and environmental health, agriculture and environmental management is crucial by placing a strong emphasis on cross-sectorial collaboration, ownership and monitoring of interventions delivery effectiveness, and supporting sustainable One Health Community of Practice and livelihoods.

The persistence of Mpox outbreak spread and dynamics revealed limited access to vaccines, diagnostic tools, health infrastructure and services disparities, exacerbated by conflict related displacement and migration, deforestation, urbanization linked human to animal encroachment in most low-resource Africa settings including DR Congo, South Africa, and Cameroon [6, 7]. These challenges and gaps hinder preparedness and containment of Mpox effectively. Also, the emergence and spread of clade Ib characterized rapid transmission, severity and containment challenges, raise both public health and animal/livestock risk. As Mpox affected countries become prepared and ready to navigate the ongoing threat, the call for equitable and sustainable accessibility, availability and uptake has become increasingly pronounced. As government, international and humanitarian agencies must prioritize quality access and uptake to at high risk marginalized communities including refugees and displaced people and livestock that are disproportionately affected by Mpox outbreak consequences [3, 7]. Thus, Mpox outbreak serves as a stark reminder of persistent vulnerabilities in Africa's public health systems and highlights the urgent need for a proactive, multifaceted approach that encompasses not only medical intervention but also education, socioeconomic reform, and global collaboration to prevent future outbreaks [3]. The lessons learned from Mpox outbreak has been instrumental in shaping a comprehensive OH emergency response strategy that builds community resilience against the reemerging infectious diseases threats across sub Saharan Africa and beyond.

Hence, prioritizing sustained and comprehensive OH interventions implementation and financing mechanism is crucial in yoking integrated human, animal and environment interface programs. This is essential to stamp out the complexity of Mpox outbreak and ensure long-term outcomes and benefits in the most vulnerable rural and remote African settings. The technical support and assistance from the Africa CDC and WHO continue to provide a significant boost by empowering local healthcare professionals and community workers to enhance early detection, timely reporting, and prompt response, including early awareness, engagement and participation. It is also important to tackle the dual threats and complexities of climate change and zoonotic outbreaks, including social stigma and mental distress that discourage individuals from seeking care [8].

## Implementation community-based one health approach in combating Mpox outbreak PHEIC in Africa

Implementing a multi-sectorial Mpox strategies for strengthening public health response and recovery interventions and actions requires a multifaceted Mpox outbreak approach involving surveillance, contact tracing, community engagement, outbreak containment, and vaccination efforts. Implementing community-based OH approaches entails nurturing robust and sustainable interdisciplinary collaboration and multisectorial coordination that promote and contribute to local ownership and participatory engagement. A better understanding of the human-animal-environment (ecosystem) health interface/dependence and governance, and quality data collection in enabling evidence-based OH outbreak decisions making and practice.

Building One Health community of practice (OH-CoP) stewardship and priorities setting is essential in fostering effective domestic and external resource mobilization strategies and sustainable financing mechanisms. Catalyzing OH-CoP impacts initiatives implementation requires efficient and transparent management, trusted and reliable risk communication channels and co-participation. Moreover, developing and implementing OH-CoP training, capacity building and educational outreach programs enabling competencies and skills acquisition and deployment students and youths, professionals, community health workers and vulnerable communities.

Investing in community-led strategic partnerships and innovative financial sourcing (e,g.: internal, external) and efficient management of OH-CoP champions and voices initiatives and increasing productivity. Optimizing OH-CoP policy and governance frameworks and structure integration into existing legislation and regulations in ensuring grassroots and community-driven human, livestock, agriculture and ecosystem long-lasting sustainability and equity.

**Fig. 1:**
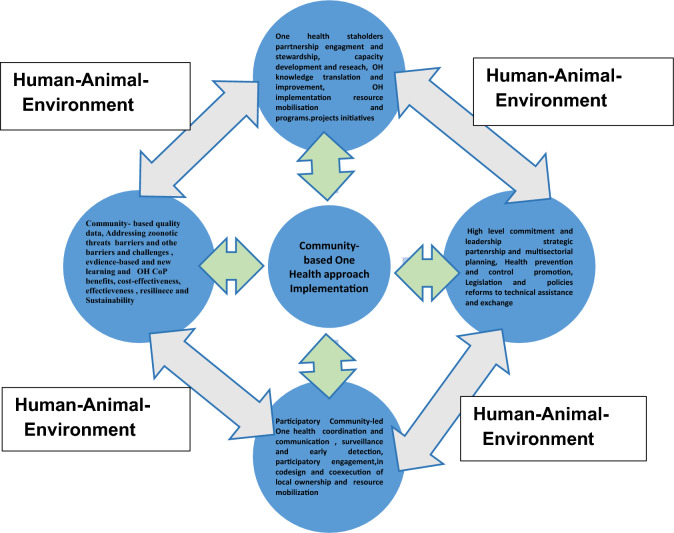
Community-based One Health programs and interventions implementation against outbreak crisis

Strengthening integrated community-based OH early warning systems can improve anticipatory and effective public health emergencies preparedness and response interventions. Also, supporting timely promotion and advocacy of outbreak emergencies humanitarian policies formulation and operationization, early risk detection and rapid reporting and coordinated strategic planning to mitigation and resilience solutions. Community-led initiatives serve to catalyze the much needed partnership for resource mobilization and community engagement inclusiveness and trust building, transparency and responsiveness, social and behavioral adaptations, maintaining best practices and resilient contingency plans management in accelerating whole-community development and empowerment during and post-outbreak emergency crisis (Fig. 1).

Leveraging on lessons learned and experiences from West Africa Ebola outbreak (2014–2015), COVID-19 (2020–2022) in tackling the growing threats and burden of emerging climate change and extreme weather sensitive infectious diseases outbreaks [2, 4, 5, 8, 9]. Such integrated and multi-sectorial strategies is necessary for strengthening emergency public health response and recovery through context-specific comprehensive and holistic public health, socioeconomic and financial response packages against Mpox outbreak to yield significant outcomes and impacts in Africa[1, 9]. There is a need to strengthen existing public health responses and lessons learned from community-based OH response against Mpox outbreak and future public health emergencies crises [7, 10].

*i. Harnessing OH approach preparedness and readiness is capital* through strengthening health systems to facilitate early detection, accurate diagnosis and sustained and equitable access and uptake of essential services and new programs including Mpox vaccination of high risk groups. Tapping from valuable experiences gained from responding to health crises to improve communities and high risk groups preparedness and readiness for future public health emergencies. It is important to analyze past preventive, cautionary and response actions and outcomes to b able identify successes, best practices, implementation gaps and address areas for improvement. This enables prioritizing early interventions, proactive approach that allows for rapid mobilization of resources and effective strategies tailored cultural and contextual emerging infectious diseases threats ou burden. Enhanced OH readiness leads to more efficient emergency responses, and minimizing public health losses [10]. Leveraging on emerging cutting-edge technologies and tools at primary healthcare and communities levels including expanding remotely digitalization and artificial intelligence-powered technologies access and deployment which offer new and continuous participation and learning, to foster a population trust and culture of resilience, essential for navigating complex public health challenges of Mpox threat and other public health emergencies today and in the future.

*ii. Strengthening community-based and primary healthcare surveillance, contact tracing and monitoring capacities* require training healthcare workers to identify Mpox cases, improve laboratory capacity for accurate diagnostics, and establish efficient reporting mechanisms.Surveillance and Medical laboratories optimization, active ports of entry or exit screening and testing travelers, sex workers and truck drivers, strengthening national disease monitoring systems and medical laboratories have been shown to substantially contribute to the increased impediment to Mpox eradication in African countries, particularly the western and central regions [11]. The disease surveillance infrastructure established by African nations during the COVID-19 pandemic is sufficient to eradicate Mpox using safe and effective vaccines, but also strengthening supply chain of medical medicines and diagnostic reagents needed to diagnose Mpox virus reservoirs and suspected cases in most African countries.

*Integrated OH contact tracing and monitoring systems* is another critical element of the response and recovery, particularly considering the human–human or animal-human transmission using dual syndromic surveillance (e.g. Thermal surveillance) and laboratory-based sequencing and real time genotyping testing confirmation [9]. For example, in Rwanda and Cameroon community health workers and health authorities were trained to effectively and efficiently performed these tasks and allowing rapid and prompt early detection and notification, tracing and quarantine advice to suspected individuals who have met confirmed cases to halt potential chains of transmission and documented Mpox clade Ib case severity management [3, 12]. Ongoing Mpox outbreak has resulted in numerous suspected and asymptomatic cases in African countries, including the DRC, Burundi, Cameroon, Rwanda and Nigeria. Although the number of reported cases in Africa was lower than in European countries and the United States, there were undoubtedly many more suspected cases and asymptomatic undiagnosed in Africa during the latest outbreak [5].

Enhancing data-driven and evidence-based policies based on building clinical and laboratory quality data, monitoring and evaluation data in promoting a robust veterinary and environmental waste biosafety and biosecurity measures and strengthening medical products supply chain reliance and impacts. These integrated public health, veterinary, environment and community based insights provide avenue for comprehensive and quality outbreak emergency preparedness packages and response impact. However, addressing challenges and gaps in financial resource mobilization, conflict related displacement and encroachment, growing demography, limited access to preventive vaccines and essential medicines, wildlife worsening the persisting vicious cycle of poverty linked inequities in the global south. For example, two countries accounts for 92% of all cases on the African continent, the DRC with approximately 80%, and highlighting the need for improved community-based OH surveillance and readiness. The DRC recorded 90% mortality of 65 cases of Mpox and only 10 laboratory-confirmed cases registered in the present outbreak [5, 13]. Furthermore, highlighting the challenges of Mpox and others emerging zoonotic diseases surveillance, preparedness and response in resource-constrained settings in most affected communities in the global south mainly in Africa.

*iii. Building Outbreak Quality Database Repository Hubs and/or Platforms Implementation for Knowledge Translation and Deployment* *is* vital in expanding and building a robust data-driven evidence policies reforms and targeted response, enhanced monitoring and evaluation systems, that is crucial for generating valuable Mpox insights and lessons learned that inform timely evidence health policies frameworks and emergency response strategies. Passive and active surveillance of Mpox outbreak continuous data collection enables identification of nature and trend, risk factors, and multifolds intervention effectiveness, and community benefits. Such data-driven approach allows public health experts to make informed decisions and adapt strategies as new information emerges. Leveraging advanced analytical tools enhances understanding of population behaviors and healthcare accessibility and medical products including Mpox vaccines availability, and optimal vaccination coverage.

**Fig. 2:**
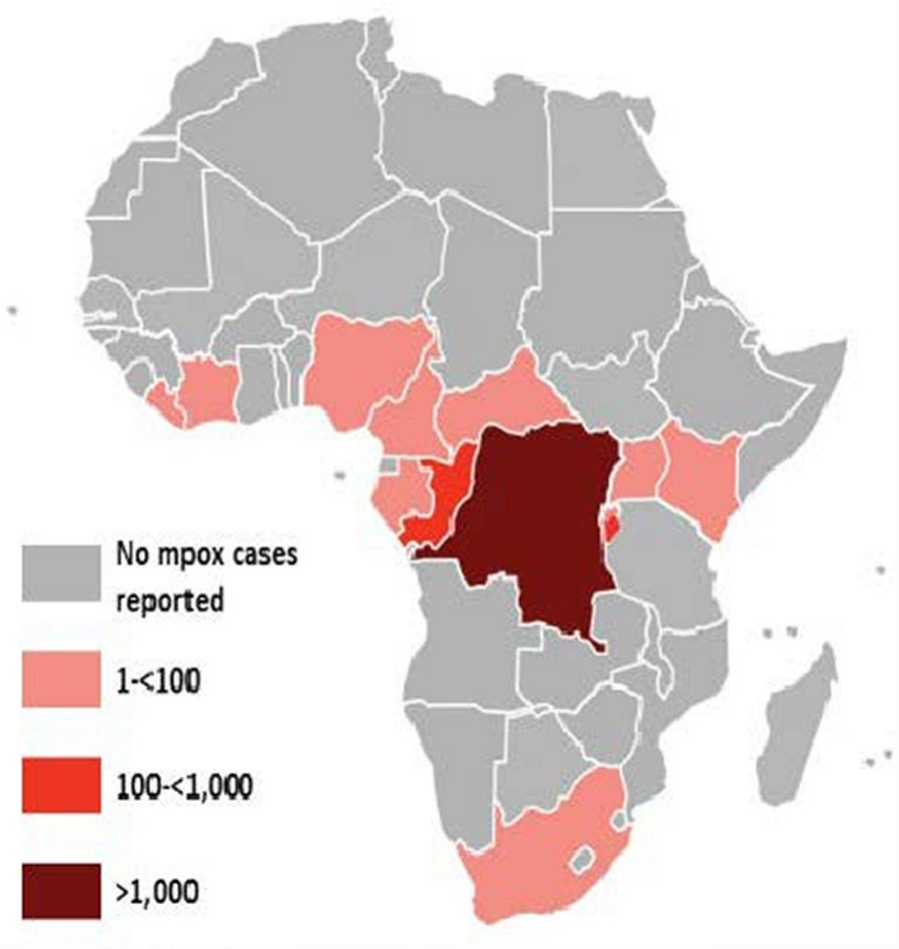
Mapping Mpox distribution of confirmed cases reported from 13 affected African countries

Fostering collaborative and participatory investment and targeted response and recovery actions leads to a more proactive and agile public health infrastructure and ensure equitable access to most effective therapies,vaccination and prevention strategies capable of swiftly addressing and quelling the current and future Mpox outbreak burden across Africa (Fig. 2).

*iv. Increased Vulnerable Communities Awareness, Educational Outreach and Trust Building Strategies* *is* needed in combating stigma and misinformation through effective public health messaging and media campaigns is vital for raising awareness about infectious diseases like Mpox outbreak. Ensuring reliable sources, transparent communication and scientifically accurate information can support vulnerable groups and public empowerment and capacities to adopt healthier behavior lifestyle adaptations, promoting care seeking culture and practices [4].

Scaling up integrated OH Mpox community awareness and health education outreach activities is crucial for at high risk groups and communities to gain a better understanding of transmission dynamics and prevention strategies. Intensifying collaboration, inclusiveness and community programs ownership by local community leaders and health district ensures the entire communities participation and set expected outcomes including cultural adapted health messages resonate and proactive response and recovery actions plans and activities across diverse populations. Promoting peer-peer educational campaigns and outreach activities have proven effective to dispel myths, social media disinformation and illed-perception surrounding Mpox, often rooted in misinformation and stigma, which can lead to societal reluctance in seeking medical care or adhere to public health measures. Ultimately, increasing community awareness and equity in Mpox vaccination safety and impact information sharing facilitates greater participation in vaccination campaigns, uptake and preventive initiatives, enhancing community resilience against Mpox outbreak and emerging infectious disease threats.

Fostering community involvement and participation in co-design and co-implementation of community-based outreach activities are vital for addressing stigma and misinformation, building mutual trust, and encouraging adherence to public health measures. Health communication strategies must be culturally sensitive and inclusive to ensure that marginalized populations receive adequate support and access to healthcare services.

*v. Scaling up Community Engagement and Risk Communication strategies* plays a vital role in this response; public health messages must be communicated in culturally sensitive manners to build trust and ensure compliance with health directives. These community-led initiatives can empower locals to take ownership of the Mpox response, promote the early reporting of symptoms, and foster productive collaboration between health authorities and communities [12]. In conjunction with vulnerable communities supportive therapies and isolation measures, quarantine protocols for confirmed and suspected cases or asymptomatic reservoir constitute a fundamental layer of response [4]. Additionally, establishing dedicated Mpox treatment centers can ensure that infected individuals receive appropriate medical care including stigma and mental health services and psychosocial support, while minimizing the risk of outbreaks in healthcare and communities settings. Investing in One Health approach Mpox outbreak containment and recovery strategies are critical, particularly in densely populated urban and remote rural poor areas including porous cross borders enhanced integrated surveillance and quarantine of human and animal suspected signs, where the risk of rapid transmission is heightened [4, 7, 14].

*vi. Intensified Vaccination Campaign and Coverage Effectiveness Efforts* particularly to health professionals, vulnerable frontline and at high risk groups (e.g.: immuno-deficient patient, Sex worker, bushmeat consumers and sellers, with the use of the JYNNEOS vaccine or a similar modified vaccine virus strain, can be instrumental in controlling the spread of Mpox [13]. Contextual and cultural adapted vaccination campaigns and educational outreach should prioritize high-risk populations, including healthcare workers, sex workers and individuals with increased exposure risk and vulnerability, as well as the surrounding communities [14]. However, understanding of cultural beliefs and practices that may impact vaccination reluctance and resistance, vaccine hesitancy and uptake, low/weak availability of vaccine and access inequity, supply chain and storage logistics challenges, including vaccine supply, distribution, and storage, must be effectively managed to ensure equitable access across diverse geographic regions [1]. Fostering global solidarity and international collaboration amongst all stakeholders, research institutions and academia exchange of knowledge and technical know-how is an asset and plays a pivotal role in promoting behaviour change for successful vaccination access and uptake, in quelling and timely lifting the Mpox outbreak PHEIC in Africa.

Effective and continuous Mpox vaccination campaigns and social media disinformation strategies coupled with robust community-based surveillance, and contact tracing are essential to significantly reduce infection or lethality rates during ongoing outbreaks in Africa and elsewhere. The implementation of these strategies limits Mpox transmission, protects vulnerable populations and timely case management using new drug Tecovirimat treatment coupled with palliative care management. It has be documented that vaccination not only provides individual immunity but also promotes herd immunity, which is crucial for community health. By closely Mpox asymptomatic surveillance and monitoring case trends and quickly isolating confirmed cases, the spread of infections can be curtailed efficiently. This comprehensive approach enhances public health outcomes and safeguards community well-being [10, 13].

*v. Leveraging Strategic Partnerships and Collaboration with global stakeholders and organizations* offers a strong and dynamic platforms for Mpox outbreak response leadership, coordination and governance, including WHO-AFRO and Africa CDC technical assistance. These are crucial for resource mobilization, technical assistance, and information sharing [5]. Strengthening international collaboration to scale local manufacturing and pharmaceutical vaccines and medical products regulatory framework, maximizing production and supply chain management to boost accessibility and update for impact in the fight against infectious diseases within Africa. In line with Africa Vision 2063”The Africa we want”, enhanced knowledge exchange and resource sharing is paramount to health, socioeconomic growth and development. A unified global outbreak preparedness and emergency response agenda and co-benefits both non-affected and affected regions is play a major role in the overall global health security sustainability [4, 6, 14].

By fostering pharmaceutical partnerships among countries, organizations, and institutions, collective expertise and resources can be leveraged with technologies advancements and innovations to address global public health challenges more effectively. Increased collaboration facilitates coordinated local and regional preparedness and response actions, ultimately leading to stamp out Mpox and better health outcomes. Hence, community based One Health interconnected approach is essential for effectively navigating and managing emerging infectious diseases/health threats that transcend national boundaries. As such, the Africa Centers for Disease Control and Prevention (Africa CDC), can facilitate a cohesive preparedness and emergency response technical support and assistance in ensuring that uniform guidelines and strategies are implemented across countries [5, 15].

The Mpox outbreak PHEIC declaration has had a significant impact on Africa and has highlighted the necessity for a coordinated response to quell similar outbreaks and preserve lives. Significant efforts have been made to ensure tight coordination with communities and governments, with country teams working on the frontlines to bolster preparedness and resilience measures to quell Mpox outbreaks. With the increasing spread of the virus, further scaling up through coordinated international efforts is essential to support African countries outbreaks/pandemics preparedness and response programs and interventions according to International Health Regulations, 2005 and global health security agenda of health equity for all [4, 7]. Fostering Africa CDC preparedness and technical capacities initiatives are crucial for Emergency Use Listing and Response for Mpox vaccinations, which potentially speed up vaccine and vaccination access and uptake for impact coverage in most remote and hard to reach communities in Africa. Emergency Use Listing also allows partners like GAVI and UNICEF partnership and support to obtain last mile access to and uptake of vaccines and vaccination impact against preventable diseases including Mpox. WHO and partners including established pharmaceutical and biotechnologies firms is collaborating with African governments on local production of vaccines and medicines by local, while strengthening manufacturing regulations and reliance harmonization for potential safe and efficacious medical products, as well as the interim Medical Countermeasures Network [7].

Furthermore, tailored community outreach activities to educate on disease prevention precautionary and control measures are dependent on correct knowledge and evidence-based findings of disease clade, transmission dynamics and severity. Laboratory and molecular assays capabilities useful in accurately awareness and educational outreach on distinct transmission routes, clinical manifestations, and afflicted groups in comparison with the 2022 pandemic [4, 16]. Public health activities must be tailored to specific local situations in order to counter mistrust, cultural differences, and disinformation. The involvement of community- and faith-based leaders, at-risk populations, and survivors is critical. As vaccine programs begin, community engagement and building trust is critical for securing the uptake and adherence to booster dosses, while limiting the spread of Mpox at high risk groups including sex workers, and immuno-compromized groups. Prioritizing One Health operationalization can increase collaboration and optimal response outcomes, while minimizing stigma, and ultimately enhance health outcomes, and satisfactory co-benefits [7]. Likewise, the recent outbreak of Mpox PHEIC by the Africa CDC and WHO owing to the possibility of international transmission and the necessity for a coordinated response actions globally. The recent detection of travel-associated clade I cases first reports outside Africa in Thailand and Sweden is indicative of the potential for clade I Mpox to spread from Africa to the rest of the world. This is, similar to the 2022 global clade II outbreak, revealing the time for global solidarity and partnership is now. Despite these ongoing country and Africa CDC efforts, the response and recovery to Mpox PHEIC in Africa remains challenging coupled with lingering community healthcare disparities, climate change and conflict political instability, and shortage of resource to persisting technical capacities gaps and consequences [4, 8, 17, 18].

*vii. Driving Public Health Response Funding Mpox outbreak* highlighted the poor response of African Union (AU) member states. Despite the seriousness of the situation, the AU and its members have a track record of under-delivery in global health security. This lack of help is aggravated by the serious economic depression that is currently afflicting many African countries, which are already under strain due to the unequal burden of debt payments [19, 20]. Owing to these limitations, the PHEIC declaration struggles to translate into effective action on the ground without significant financial commitments or political will from member states [4]. This lesson highlights the need for financing support from multiple organizations to help drive public health responses. As a result, the WHO forecasts an initial budget requirement of $15 million to support surveillance, preparedness, and response efforts in Africa [8, 16, 21]. However, to allow for an urgent scale-up, the WHO has approved $1.45 million from the WHO Contingency Fund for Emergencies, with a greater need to combat the outbreak.

Strengthening local and country emergency One Health preparedness and response, as recommended by Africa CDC and WHO should be prioritize as funding Mpox response and recovery programs. African CDC and WHO have enabled scaling up of the response capacities to the outbreak, which has so far been underwhelming because of inadequate budget commitments of $1 million from WHO and $10.4 million and might require larger funds to contain the ongoing outbreak in African Union members countries [4, 6, 21, 22]. Investing on One Health community of practice is necessary in deepening the vulnerable people knowledge, bridging multidisciplinary interactions and fostering multisectorial collaboration and data-driven decisions making,and inspired new contextual ideas and approaches in accelerating social transformation.

*viii. Improved OH Community Resilience and Equity* is crucial in strengthening the health infrastructure access and update of health commodities through comprehensive geographical access and training, enhanced diagnostic capabilities, and systemic resource mobilization to significantly bolster the community healthcare resilience and participatory initiatives. More resilient community systems and activities including local engagement in decision making, shared resources, inclusiveness and equitable allocation of resource are needed to address outbreak related health inequities. Also, addressing the social and behavioural determinants (e.g.: stigma, trust, knowledge, adherence) of health is essential in enhancing resilience and survival champions against Mpox outbreaks, which often are disproportionately marginalized within affected communities [4, 19, 24, 23]. Furthermore, stigma and discrimination associated with Mpox infection can be conquered through psychosocial support programs targeting the most vulnerable groups is critical; as the historical context of the disease has led to misconceptions that can deter individuals from seeking care or reporting their conditions. Hence, OH community-based and primary healthcare approaches that merge human and animal clinical, environment and sociocultural insights are necessary for effective intervention [2, 4, 16, 23]. In conclusion, the public health response to Mpox in Africa must be comprehensive and adaptive, incorporating surveillance, contact tracing, community engagement, outbreak containment, and vaccination as the key components. Despite facing formidable challenges, concerted efforts to mobilize health authorities, communities, and international partners have the potential to mitigate the impact of Mpox outbreaks effectively [24–26].

The lessons learned from previous Ebola outbreaks and COVID-19 pandemic are invaluable assets in shaping future community and public health emergency responses to infectious disease threats, and underscoring the importance of OH approach community preparedness and resilience for health equity for all and upholding global health security across Africa [3, 6, 27, 28]. Sustained government and multi-stakeholders commitment and financial investment remain crucial to strengthening community and primary healthcare infrastructures, precautionary and preventive packages in addressing socio-cultural and behavioural determinants of health during crises. Scaling community-based OH approach is core to improve primary healthcare preparedness and readiness strategies to effectively detect, prevent, respond and manage future outbreaks and crises [18, 29]. It also requires establishing robust and sustained early warning health system to ensure early warning alerts and readiness to respond rapidly to local and global health emergencies such West Africa Ebola outbreak and COVID-19 pandemics [26]. Investment in integrated OH resilience measures will ultimately safeguard the health and well-being of African vulnerable communities and building a stronger foundation of health systems to better withstand the unprecedented challenges posed by re-emerging and reemerging infectious diseases threats and burden [4, 24, 28, 30, 31]. Scaling up equitable access to primary healthcare and ample resource allocation for targeted Mpox vaccination efforts are essential to ensure that marginalized and high-risk communities receiving equitable access to healthcare services[22], improves community health outcomes and livelihoods in tackling health disparities those often exacerbate the impact of Mpox outbreaks [4, 22, 29, 32, 33].

## Conclusions

Community-based OH implementation offers tremendous opportunities and paths to more community resilience and sustainable human, animal and environmental coordinated, collaborative and committed local leadership in preparedness and emergency response actions against zoonotic outbreaks threats and burden in Africa. This highlights the critical need for a strengthening regional OH preparedness and emergency response to combat zoonotic outbreaks effectively and ensure that no community is left behind. Moreover, addressing OH approach implementation against climate sensitive diseases outbreaks and inter-operatibility challenges underline the necessity of developing comprehensive, adaptive, and equitable OH community and public health frameworks, governance and co-benefits implementation paths in Africa. By focusing on community-based OH early warning surveillance and monitoring systems, country can enhance its integrated resilience to ongoing and future infectious disease threats. Promoting OH biosafety and hygiene practice in both clinical and environmental settings, early warning alerts, surveillance and response systems, indicators and evaluation metrics those are vital in preventing and controlling Mpox and others outbreak, enhancing risk communication and culturally-sensitive education outreach activities in all remote and vulnerable communities [4, 9, 27, 33]. Harnessing human and wildlife/livestock-human transmission monitoring, reducing deforestation and encroachment linked environmental stewardship long-term strategies in high risk countries. The key OH Mpox preparedness and response lessons learned emphasize the importance of adequate funding, enhanced OH surveillance and timely diagnostic capacities, and robust community engagement in contact tracing and effective case management [5]. Better understanding of sustained financial support and financial mechanisms are essential for improving health infrastructure and emergency preparedness, enabling swift resource mobilization and effective action against outbreaks. Strengthening laboratory diagnostics and real-time data-driven OH preparedness and response interventions outcomes and impacts against bio-threats public health emergencies responses.

## Data Availability

All datasets generated or analyzed the current study are available.

## Abbreviations

WHO: World Health Organization
Africa CDC: Africa Centre for Disease Control
Mpox: Monkeypox
PHEIC: Public health emergency of international concern
WASH: Water, hygiene and sanitation
CFR: Case fatality rate
GAVI-Gavi: The vaccine alliance
UNICEF: The United Nations children’s fund
COVID-19: Coronavirus disease 2019
